# Epidemiology of Human T-cell Lymphotropic Virus Type 1 Infection in Blood Donors, Israel

**DOI:** 10.3201/eid1507.080796

**Published:** 2009-07

**Keywords:** **Keywords:** HTLV-1 infections, human T-cell lymphotropic virus type 1, viruses, epidemiology, Israel, emigration and immigration, blood banks, Middle East, dispatch

## Abstract

The prevalence of infection with human T-cell lymphotropic virus type 1 (HTLV-1) in blood donors from Israel is 1 infection/100,000 persons. In donors originating from Eastern Europe, the Middle East, and Latin America, prevalences are 7.7, 14.6, and 20.4, respectively. HTLV-1 prevalence may be high outside areas where HTLV-1 previously was known to be endemic.

Human T-cell lymphotropic virus type 1 (HTLV-1) is prevalent mostly in Japan, Africa, the Caribbean Islands, and South America (*1**,**2*). Known HTLV-1 modes of transmission include vertical transmission (predominantly through breastfeeding), transverse transmission (sexual intercourse), transfusion of infected cellular blood products, and sharing of needles and syringes (*1**,**2*). Because of reports of HTLV-1–associated diseases in Mashhadi Jews, the Israeli national blood services, Magen David Adom, began screening all blood units for HTLV-1 antibodies in 1995. However, the prevalence of HTLV-1 infection in the general Israeli population has not yet been defined.

Israel is an immigration state, providing a unique opportunity to examine the prevalence of HTLV-1 infection according to donors’ countries of origin. This information may reflect the distribution of HTLV-1 within the respective countries of origin, some of which have not had HTLV-1 serosurveys performed.

## The Study

Blood donation in Israel is voluntary and does not involve any monetary benefit. Using records from Magen David Adom, we registered age, sex, country of birth, and maternal and paternal countries of birth once for each donor, regardless of the number of blood units donated.

From 1995 through 1998, donors were screened for antibodies against HTLV-1 and HTLV-2 by standard ELISA (Abbott HTLV-1/HTLV-2 enzyme immunoassay; Abbott Laboratories, Abbott Park, IL, USA). Since 1998, testing has been performed by chemiluminescent immunoassay with the PRISM assay (Abbott Laboratories). The confirmatory assay was Western blot HTLV Blot 2.4 (Genelabs Diagnostics, Singapore Science Park, Singapore).

**Figure 1:**
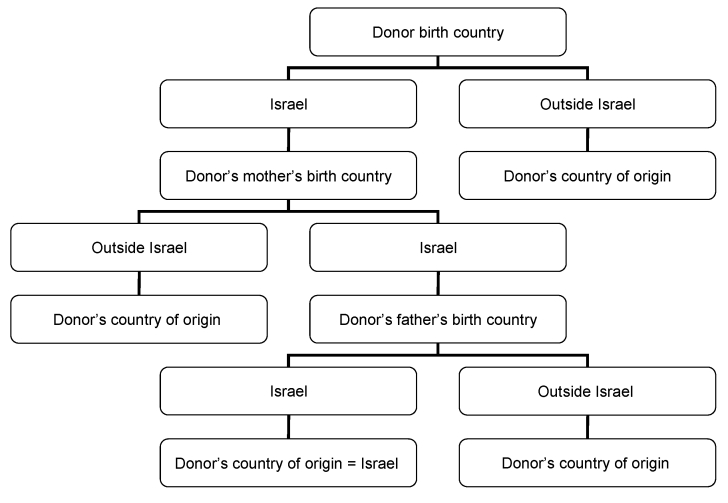
Algorithm for identifying the geographic origin of Israeli blood donors.

On the basis of virus transmission modes, we developed an algorithm for identifying the ethnic origin of both HTLV-1–positive and HTLV-1–negative blood donors (Figure 1). We considered infection to be acquired in Israel when the donor and both parents were born in Israel. We considered infection to be acquired outside Israel when the donor or 1 parent was born outside Israel. When the donor was born in Israel and the mother was born outside Israel, country of origin was considered the mother's country of birth. When the donor and the mother were born in Israel, but the father was born outside Israel, country of origin was considered the father’s country of birth. Detailed classification of geographic origin of blood donors (both HTLV-1 positive and HTLV-1 negative) is given in the Technical Appendix.

Data were analyzed by using Microsoft Access (Microsoft, Redmond, WA, USA) and Epi Info (Centers for Disease Control and Prevention, Atlanta, GA, USA); statistical analysis was conducted by using χ^2^ analysis of contingency tables. The odds ratio (OR) and 95% confidence interval were calculated. Age was described as mean ± standard deviation. The Chaim Sheba Medical Center human subjects research review board approved this study.

From January 9, 1995, through December 31, 2003, a total of 1,256,669 blood donors were screened for HTLV-1 infection in Israel. Of these, 73 HTLV-1 carriers were identified, for an overall prevalence of 5.8 infections per 100,000 donors. Average age at diagnosis was 39.4 ± 11.9 years; 48 (66%) were men (compared with 72% of all blood donors; p = 0.3125). All HTLV-1–positive donors had negative serologic results for HTLV-2, human immunodeficiency virus, hepatitis C virus, and hepatitis B surface antigen. HTLV-1 carriers originated from 20 countries (Table).

**Table:** Prevalence of HTLV-1 in blood donors from different countries of origin, Israel, 1995–1998*

Country	No. HTLV-1 carriers	No. blood bank donors	No. carriers/ 100,000 donors
Iran	16	31,776	50.4
Romania	12	73,971	14.9
Iraq	7	68,857	10.2
Russian Federation	7	111,109	6.3
Turkey	4	25,054	16
Poland	3	70,172	4.3
Israel	3	294,342	1.0
Morocco	3	144,014	2.1
United States	3	49,204	6.1
Yugoslavia†	2	3,181	62.9
Uruguay	2	3,552	56.3
Argentina	2	20,898	9.6
Chile	2	2,101	95.2
Czechoslovakia†	1	11,149	9.0
Brazil	1	4,217	23.7
Niger	1	1	
Ethiopia	1	3,412	29.3
Egypt	1	21,245	4.7
Yemen	1	36,052	2.8
Libya	1	21,427	4.7

*HTLV-1, human T-lymphotropic virus type 1. †Country no longer exists.

**Figure 2:**
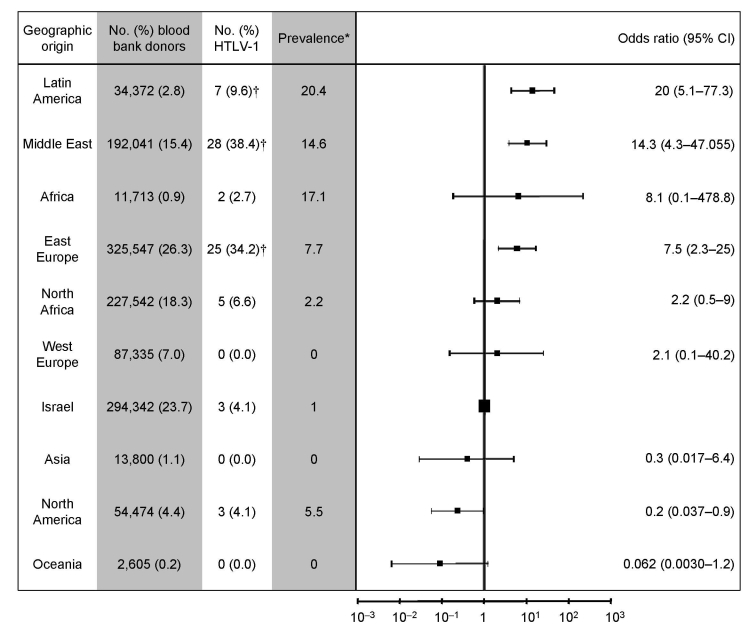
Relative risk for human T-cell lymphotropic virus type 1 carriage in donors from different geographic origins. Odds ratios (indicated by black boxes) are charted in logarithmic scale. Error bars indicate 95% confidence intervals (CI). *Per 100,000 population; †p<0.05.

ORs for HTLV-1 carriers varied by geographic origin of donor (Figure 2). Donors from Middle Eastern and Eastern European countries were at highest risk for HTLV-1 carriage.

## Conclusions

The diversity of the population in Israel, combined with systematic screening of blood donors, enabled us to examine the global epidemiology of HTLV-1 infection. One or both parents of at least 67% of Jews in Israel were born outside Israel. Thirteen percent immigrated from Asia, 16% from Africa, and 36% from Europe and the Americas (*3*). We believe that blood donors are representative of healthy persons in the general population of the country because approximately one third of the population are blood donors.

The prevalence of HTLV-1 infection among donors in Israel was 5.8/100,000 but varied depending on donors’ geographic origins. In blood donors born in Israel, the prevalence of HTLV-1 infection was 1/100,000, comparable to and even lower than the prevalence of HTLV-1 in blood donors from northern Europe (Technical Appendix). Unfortunately, information from many countries is either lacking or unrepresentative. We found a similar prevalence of HTLV-1 infection in Jewish immigrants from known HTLV-1 endemic countries to what is known in their native countries. For example, the reported prevalence in blood donors in Latin American countries ranged from 30/100,000 to 1,000/100,000 (Technical Appendix); our study found a prevalence of 20/100,000.

In donors from the Middle East, we found a high prevalence of HTLV-1 infection in blood donors from Iran (50.4/100,000), Turkey (16.0/100,000), and Iraq (10.2/100,000). Of those countries, only Iran can provide information about HTLV-1 prevalence, and those data are limited to Mashhad (*4*). Our survey was performed after the initial report of HTLV-1–associated disorders in Israelis born in or originating from Mashhad (*5*). This example can highlight the role of immigrants as reflectors of the populations in their countries of origin. We found no systematic information about the epidemiology of HTLV-1 in the rest of Iran and neighboring countries. However, anecdotal reports suggest that the infection exists in Kuwait, Turkmenistan, and Georgia (*6**–**8*).

In persons from eastern Europe, we found a high prevalence of HTLV-1 carriage in blood donors originating from Romania (14.9/100,000), Russia (6.3/100,000), and the former Yugoslavia (62.9/100,000). Most of the literature does not consider eastern Europe endemic for HTLV-1 infection (*2*). Particularly in Romania, HTLV-1 prevalence is considered low, and HTLV-1 positivity was attributed mainly to immigration from other countries (*2*). In a careful review of the literature, we found only a few studies involving a small number of blood donors (Technical Appendix). These studies support our findings and suggest that HTLV-1 is endemic in some eastern European countries. Several cases of adult T-cell leukemia have been described in patients of Romanian origin (*9**–**14*).

Our findings establish the need for rigorous screening of blood donors in suspected HTLV-1–endemic areas (Middle East and eastern Europe) and show the impact of immigrants or travelers as sentinels to the health problem in their country of origin. Our data also demonstrate high prevalence of HTLV-1 infection among those whose country of origin had been considered nonendemic for HTLV-1. Viral transmission could have occurred either from the non-Jewish local population or from other Jewish communities in which HTLV-1 is endemic. We are not aware of immigration waves of Jewish populations from Iran and Iraq to Eastern Europe and vice versa. In addition, the prevalence of HTLV-1 in blood donors from Israel is low. Therefore, it seems reasonable to conclude that our findings reflect local HTLV-1 endemicity in the country of origin.

## Supplementary Material

Technical Appendix Classification of Countries for Determination of Ethnic Origin
