# Supplementary material for: Epidemiology of Human T-cell Lymphotropic Virus Type 1 Infection in Blood Donors, Israel
Source: Emerg Infect Dis. 2009 Jul;15(7):1116–8. doi: 10.3201/eid1507.080796 (PMC2744246; doi:10.3201/eid1507.080796)
Supplement: Technical Appendix — Classification of Countries for Determination of Ethnic Origin [file 08-0796_Techapp-s1.pdf]

DOI: 10.3201/eid1507.080796

Epidemiology of Human T-cell Lymphotropic Virus Type 1 Infection in Blood Donors, Israel

Technical Appendix

Classification of Countries for Determination of Ethnic Origin

Asia

Bangladesh, Bhutan, Cambodia, People's Republic of China, Hong Kong, India, Indonesia, Japan, Republic of Korea, Lao People's Democratic Republic, Malaysia, Mongolia, Myanmar, Nepal, Pakistan, the Philippines, Singapore, Sri Lanka, Taiwan, People's Republic of China, Thailand, Vietnam

Central/South America

Argentina, Belize, Bolivia, Brazil, Chile, Colombia, Costa Rica, Cuba, Dominica, Ecuador, El Salvador, French Guiana, Guatemala, Guyana, Haiti, Honduras, Jamaica, Mexico, Panama, Paraguay, Peru, Puerto Rico, Suriname, Trinidad and Tobago, Uruguay, Venezuela

Eastern Europe

Albania, Belarus, Bulgaria, Czechoslovakia (no longer exists), Estonia, Hungary, Latvia, Lithuania, Republic of Moldova, Poland, Romania, Russian Federation, Ukraine, Union of Soviet Socialist Republics (no longer exists), Yugoslavia (no longer exists)

Western Europe

Austria, Belgium, Denmark, Finland, France, Germany, Greenland, Iceland, Ireland, Italy, the Netherlands, Norway, Portugal, Spain, Sweden, Switzerland, United Kingdom (Great Britain).

Middle East

Afghanistan, Armenia, Azerbaijan, Cyprus, Georgia, Greece, Iraq, Islamic Republic of Iran, Jordan, Kazakhstan, Kuwait, Kyrgyzstan, Lebanon, Saudi Arabia, Syrian Arab Republic, Tajikistan, Turkey, Turkmenistan, Uzbekistan, Yemen

North Africa

Algeria, Egypt, Libyan Arab Jamahiriya, Morocco, Sudan, Tunisia

North America

Canada, United States of America

Oceania

Australia, New Zealand, Papua New Guinea

Sub-Saharan Africa

Angola, Benin, Burkina Faso, Burundi, Cameroon, Central African Republic, Chad, Republic of Congo, Côte d'Ivoire (Ivory Coast), Equatorial Guinea, Ethiopia, Gabon, Ghana, Guinea, Guinea-Bissau, Kenya, Liberia, Madagascar, Malawi, Mali, Mauritania, Mozambique, Somalia, Sierra Leone, Senegal, Rwanda, Nigeria, Niger, Nepal, Namibia, United Republic of Tanzania, Togo, Uganda, Zaire, Zambia, Zimbabwe

Technical Appendix Table. Worldwide prevalence of human T-cell lymphotropic virus 1 (HTLV-1) carriers among blood donors*

Region and location of study population	No. donors tested	No. HTLV-1–positive donors	Prevalence/100,000 population	Ref.
Eastern Europe				
Latvia	150	0	0	(*1*)
Romania	621	4	640	(*2*)
Russia, Moscow†	NA	NA	1,400	(*3*)
Western Europe				
Denmark	NA	NA	2	(*4*)
Europeans without obvious risk factors	NA	NA	1–2	(*4*)
France	NA	NA	7	(*4*)
France	1,816,927	66	3.9	(*5*)
The Netherlands	674,000	15	2	(*4*)
Southwestern Greece	107,302	NA	5.6	(*6*)
Spain	530,000	NA	1.1	(*7*)
Sweden	235,000	6 (countries of origin: Sweden, 3; Denmark, 1; UK, 1; Iran, 1; Chile, 1)	2 (crude; 1 if born in Sweden, 1.3 if born in Europe)	(*4*)
Sweden	612,000	6	1	(*8*)
Italy	68,520	5	7	(*8*)
United Kingdom	NA	NA	5	(*4*)
North America				
Canada‡	81,962	10	12.2	(*9*)
USA	39,898	10	25	(*10*)
USA‡	NA	NA	9.6	(*11*)
USA	6,400,000	442	7	(*8*)
Latin America				
Argentina	76 246	25	33	(*12*)
Argentina, Buenos Aires	86,238	NA	34	(*13*)
Brazil	6,218,619	NA	40–1,000	(*14*)
Caracas, Venezuela	23 413	23	98	(*15*)
Middle East				
Iran, Mashhad	28,928	208	770	(*16*)
Saudi Arabia	16,434	9	40	(*17*)
Lebanon	1,900	0	0	(*18*)
Turkmenistan	1,510	3	199	(*19*)
North Africa				
Egypt	133	3	2,255	(*20*)
Tunisia	500	1	50	(*21*)
Sub-Saharan Africa				
Burkina Faso	191	2	1,047	(*22*)
Senegal	4,900	7	143	(*23*)
Asia				
India§	10,000	18	180	(*24*)
Russia, Komsomol'sk-na-Amure†	NA	NA	1,600	(*3*)
Russia, Yuzhno-Sakhalinsk†	NA	NA	5,450	(*3*)

*Ref., reference; NA, not available.
†By passive agglutination.
‡By ELISA.
§Combined HTLV-1 and HTLV-2.

References

1. Kozireva S, Nemceva G, Danilane I, Pavlova O, Blomberg J, Murovska M. Prevalence of blood-borne viral infections (cytomegalovirus, human herpesvirus-6, human herpesvirus-7, human herpesvirus-8, human T-cell lymphotropic virus-I/II, human retrovirus-5) among blood donors in Latvia. Ann Hematol. 2001;80:669–73. Medline DOI: 10.1007/s002770100359

2. Paun L, Ispas O, Del Mistro A, Chieco-Bianchi L. HTLV-I in Romania. Eur J Haematol. 1994;52:117–8. Medline

3. Seniuta NB, Iakovleva LS, Stepina VN, Buachidze LN, Gurova EP, Kologrivova ZA, et al. [Screening of sera from the adult populations of some USSR regions for antibodies to the human T-cell leukemia virus type I (HTLV-I)]. Vopr Virusol. 1990;35:309–12. Medline

4. Tynell E, Andersson S, Lithander E, Arneborn M, Blomberg J, Hansson HB, et al. Screening for human T cell leukaemia/lymphoma virus among blood donors in Sweden: cost effectiveness analysis. BMJ. 1998;316:1417–22. Medline

5. Courouce AM, Pillonel J, Lemaire JM, Maniez M, Brunet JB. Seroepidemiology of HTLV-I/II in universal screening of blood donations in France. AIDS. 1993;7:841–7. Medline

6. Tseliou PM, Spanakis N, Spiliotakara A, Politis C, Legakis NJ, Tsakris A. HTLV-I and -II in southwestern Greece. Transfusion. 2003;43:1641–2. Medline DOI: 10.1046/j.1537-2995.2003.00569.x

7. Toro C, Rodes B, Aguilera A, Caballero E, Benito R, Tuset C, et al. Clinical impact of HTLV-1 infection in Spain: implications for public health and mandatory screening. J Acquir Immune Defic Syndr. 2002;30:366–8. Medline

8. Courouce AM, Pillonel J, Saura C. Screening of blood donations for HTLV-I/II. Transfus Med Rev. 1999;13:267–74. Medline DOI: 10.1016/S0887-7963(99)80057-1

9. O'Brien SF, Yi Q-L, Fan W, Scalia V, Kleinman SH, Vamvakas EC. Current incidence and estimated residual risk of transfusion-transmitted infections in donations made to Canadian Blood Services. Transfusion. 2007;47:316–25. Medline DOI: 10.1111/j.1537-2995.2007.01108.x

10. Williams AE, Fang CT, Slamon DJ, Poiesz BJ, Sandler SG, Darr WF, et al. Seroprevalence and epidemiological correlates of HTLV-I infection in U.S. blood donors. Science. 1988;240:643–6. Medline DOI: 10.1126/science.2896386

11. Dodd RY, Notari EP, Stramer SL. Current prevalence and incidence of infectious disease markers and estimated window-period risk in the American Red Cross blood donor population. Transfusion. 2002;42:975–9. Medline DOI: 10.1046/j.1537-2995.2002.00174.x

12. Remesar M, Mangano A, Sen L, del Pozo A. Profile of human T-cell lymphocytotrophic virus-I/II infections in an Argentinean blood bank population. Vox Sang. 2002;83:364–5. Medline DOI: 10.1046/j.1423-0410.2002.00240.x

13. Mangano AM, Remesar M, del Pozo A, Sen L. Human T lymphotropic virus types I and II proviral sequences in Argentinian blood donors with indeterminate Western blot patterns. J Med Virol. 2004;74:323–7. Medline DOI: 10.1002/jmv.20172

14. Catalan-Soares B, Carneiro-Proietti AB, Proietti FA. Heterogeneous geographic distribution of human T-cell lymphotropic viruses I and II (HTLV-I/II): serological screening prevalence rates in blood donors from large urban areas in Brazil. Cad Saude Publica. 2005;21:926–31. Medline DOI: 10.1590/S0102-311X2005000300027

15. Leon G, Quiros AM, Lopez JL, Hung M, Diaz AM, Goncalves J, et al. [Seropositivity for human T-lymphotropic virus types I and II among donors at the Municipal Blood Bank of Caracas and associated risk factors]. Rev Panam Salud Publica. 2003;13:117–23. Medline DOI: 10.1590/S1020-49892003000200012

16. Abbaszadegan MR, Gholamin M, Tabatabaee A, Farid R, Houshmand M, Abbaszadegan M. Prevalence of human T-lymphotropic virus type 1 among blood donors from Mashhad, Iran. J Clin Microbiol. 2003;41:2593–5. Medline DOI: 10.1128/JCM.41.6.2593-2595.2003

17. Taha MA, Bashawri LA, Ahmed MS, Ahmed MA. Prevalence of antibodies to human T-lymphotropic viruses types I and II among healthy blood donors. Saudi Med J. 2003;24:637–40. Medline

18. Naman R, Klayme S, Naboulsi M, Mokhbat J, Jradi O, Ramia S. HTLV-I and HTLV-II infections in volunteer blood donors and high-risk groups in Lebanon. J Infect. 2002;45:29–31. Medline DOI: 10.1053/jinf.2002.1006

19. Senyuta N, Syrtsev A, Yamashita M, Stepina V, Susova O, Scherbak L, et al. Sero-epidemiologic and phylogenetic studies of HTLV-I infection in 2 countries of the Caspian Sea region. Int J Cancer. 1998;77:488–93. Medline DOI: 10.1002/(SICI)1097-0215(19980812)77:4<488::AID-IJC2>3.0.CO;2-Q

20. Constantine NT, Fathi Sheba M, Corwin AL, Danahy RS, Callahan JD, Watts DM. A serosurvey for HTLV-I among high-risk populations and normal adults in Egypt. Epidemiol Infect. 1991;107:429–33. Medline DOI: 10.1017/S0950268800049074

21. Mojaat N, Kaabi H, Hmida S, Maamar M, Slama S, Boukef K. Seroprevalence of HTLV-I/II antibodies in blood donors and different groups at risk in Tunisia. J Acquir Immune Defic Syndr. 1999;22:314–5. Medline

22. Collenberg E, Ouedraogo T, Ganame J, Fickenscher H, Kynast-Wolf G, Becher H, et al. Seroprevalence of six different viruses among pregnant women and blood donors in rural and urban Burkina Faso: A comparative analysis. J Med Virol. 2006;78:683–92. Medline DOI: 10.1002/jmv.20593

23. Diop S, Calattini S, Abah-Dakou J, Thiam D, Diakhate L, Gessain A. Seroprevalence and molecular epidemiology of human T-Cell leukemia virus type 1 (HTLV-1) and HTLV-2 in blood donors from Dakar, Senegal. J Clin Microbiol. 2006;44:1550–4. Medline DOI: 10.1128/JCM.44.4.1550-1554.2006

24. Kumar H, Gupta PK. Is seroprevalence of HTLV-I/II among blood donors in India relevant? Indian J Pathol Microbiol. 2006;49:532–4. Medline
